# Decoding Cdk1 control: from mitotic thresholds to meiotic specificity

**DOI:** 10.1007/s10577-026-09796-4

**Published:** 2026-03-12

**Authors:** Sandra A Touati

**Affiliations:** Institut Jacques Monod, Université Paris Cité, CNRS, F-75013 Paris, France

**Keywords:** Mitosis, Meiosis, Cdk1, Shokat, Phosphoproteome

## Abstract

The eukaryotic cell cycle is one of the most fundamental biological processes, ensuring the accurate duplication and segregation of the genome during mitosis. Decades of research across model systems have shown that this process is orchestrated by a family of protein kinases known as cyclin-dependent kinases (Cdks). Together with their cyclin partners, Cdks act as master regulators of cell division, coordinating DNA replication, chromosome segregation, and cytokinesis with remarkable precision. The discovery of Cdks and cyclins in yeast and sea urchins, celebrated with the Nobel Prize of Hartwell, Hunt, and Nurse (awarded in 2001), established the conceptual framework for understanding how oscillations in kinase activities drive cell cycle progression in a unidirectional and irreversible manner. Over the past thirty years, a central question has been whether cell cycle control relies primarily on the quantitative level of Cdk1 activity or whether distinct qualitative functions of cyclin-Cdk1 complexes ensure the correct ordering of events. Addressing this question required new genetic and biochemical tools capable of controlling Cdk1 activity with high temporal resolution and specificity. A turning point came in 2000 with the development of the analogue-sensitive Cdk1 allele by the Shokat laboratory. This approach replaced classical temperature-sensitive alleles with a version of Cdk1 that can be selectively inhibited by bulky ATP analogues. Beyond specific inhibition, the system was soon adapted to directly label and identify Cdk1 substrates, coupling chemical genetics with the emerging power of mass spectrometry. This review outlines the conceptual frameworks of quantitative and qualitative models of Cdk1 control. It also highlights how these ideas have been experimentally dissected, tracing the development of the Cdk1 Shokat system and advances from synthetic biology and phosphoproteomics in decoding phosphorylation logic, and how these concepts apply to meiosis. These studies draw primarily on budding yeast and fission yeast which have a single Cdk, making them convenient models for studying core principles of cell cycle regulation. Key insights from vertebrates are also integrated to illustrate principles that extend to other eukaryotes.

## Mitotic Cdk1 control

The temporal separation of DNA replication in S phase and cell division in M phase is essential to ensure complete genome duplication before chromosome segregation. The unidirectionality of the cell cycle is maintained by at least four parameters: the progressive increase and decrease of Cdk1 activity, the succession of different cyclin-Cdk1 complexes throughout the cycle to confer substrate specificity, the proteasome-dependent degradation of key regulators at precise times, and checkpoints that verify the completion of critical steps. Cdks, in association with cyclins to form cyclin-Cdk complexes, are the main regulators of the cell cycle. Although mammals possess many Cdks, only one Cdk controls cell cycle progression in budding and fission yeasts (Nurse 2000; Bloom and Cross 2007; Morgan 2007).

### Decoding Cdk1: lessons from the Shokat approach

In eucaryotes, Cdks are proline-directed kinases that phosphorylate serine and threonine residues (SP/TP). These phosphorylations are reversible because because they can be removed by phosphatases. The dynamic interplay of phosphorylation and dephosphorylation activates, inactivates, or targets proteins for degradation, thereby orchestrating cell cycle events (Morgan 2007; Sullivan and Morgan 2007).

Cdk1 structure consists of two lobes. The small N-terminal lobe is composed of β-sheets with a glycine-rich inhibitory element, the G-loop (also called P-loop), which is involved in ATP binding, and a C-helix containing the conserved PSTAIRE sequence, essential for cyclin binding. The large C-terminal lobe contains the catalytic pocket and the T-loop (activation loop), which carries the activating threonine residue (T169 in budding yeast; T161 in human Cdk1; T160 in Cdk2). ATP binds at the interface of the two lobes, coordinated by the G-loop and conserved motifs in the closely positioned catalytic cleft. In the absence of cyclins, Cdk1 is inactive because the PSTAIRE helix is mispositioned and the T-loop partially blocks the catalytic pocket. Cyclin binding and T169 phosphorylation reposition the T-loop, open the catalytic cleft, and allow substrate phosphorylation (Jeffrey et al. 1995; Pavletich 1999; Echalier et al. 2014; Brown et al. 2015).

**Fig. 1:**
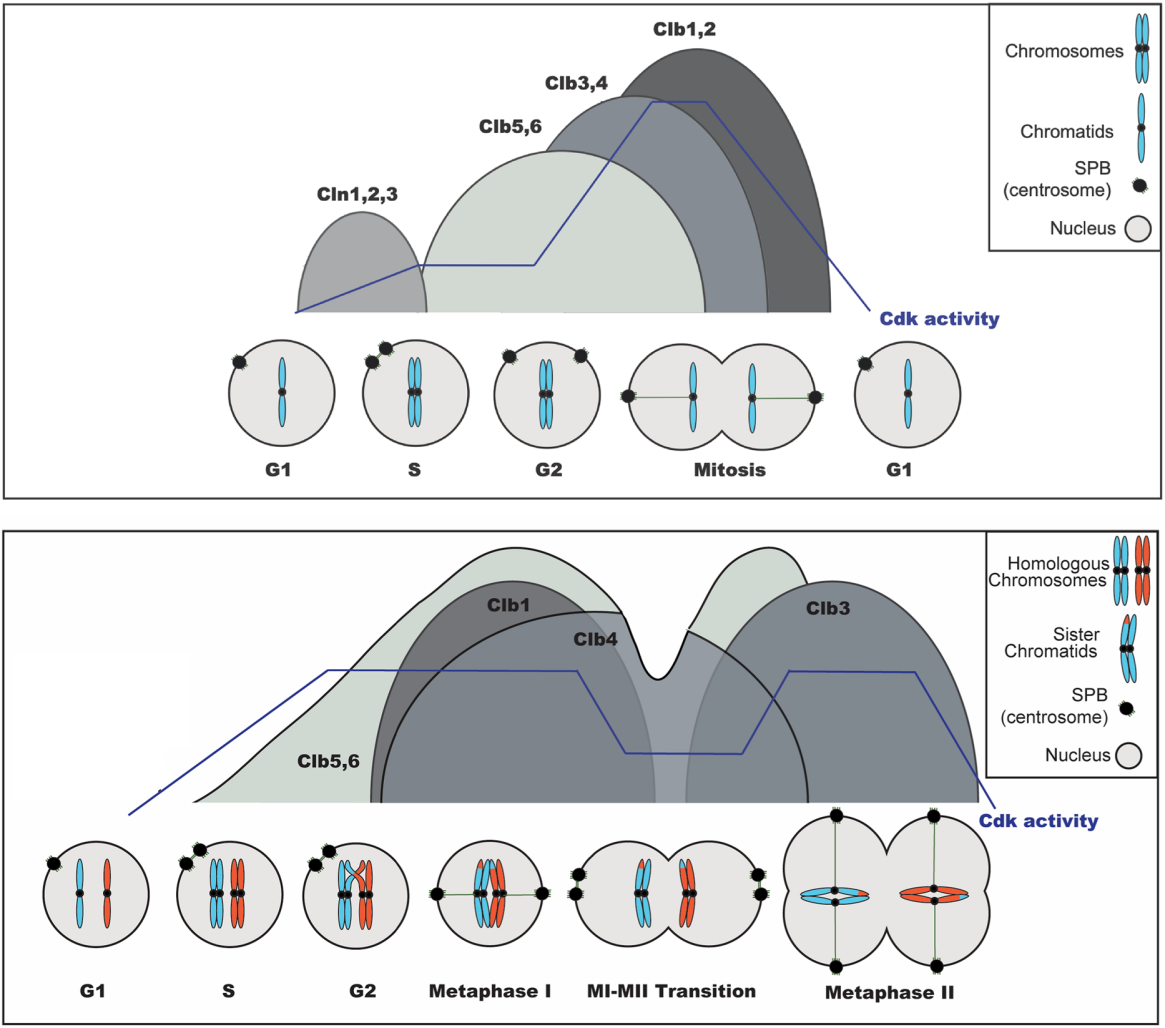
Cyclin–Cdk activity waves during the mitotic (top) and meiotic (bottom) cell cycles. Schematic representation of the rise and fall of Cyclin-Cdk activity driving successive cell cycle transitions. Mitotic activity curves are adapted from the works of the Nurse, Morgan, and Loog laboratories, whereas the meiotic activity profile is based on studies from the Amon laboratory. Theoretical global Cdk1 activity profiles are illustrated accordingly

In budding yeast, a single Cdk (Cdc28^Cdk1^) binds one of nine cyclins (three Clns and six Clbs), which function depending on the phase of the cell cycle. Cln1-3 are G1 cyclins, Clb5-6 are S-phase cyclins, Clb3-4 are G2 cyclins, and Clb1-2 are M-phase cyclins. Cyclins recognize substrates through specific docking motifs, binding to short linear motifs (SLiMs) present on their target proteins (Fig. 1). Different cyclin-Cdk1 complexes target distinct substrates in S or M phase, ensuring ordered cell cycle progression. Cyclins are expressed in waves and degraded by the proteasome. They are targeted for degradation by two E3 ubiquitin ligases, depending on the cell cycle phase: the SCF (Skp1 Cullin F-box protein complex) is active in interphase, whereas the APC/C (Anaphase-Promoting Complex/Cyclosome) functions in mitosis. Moreover, APC/C core subunits bind to two co-factors for activity and specificity named Cdc20 or Cdh1. APC/C^Cdc20^ triggers degradation at the metaphase-anaphase transition, while APC/C^Cdh1^ becomes active only after Cdk1 activity declines in anaphase and remains active until G1/S. Overall, Cdk1 activity increases progressively from G1 to metaphase through successive waves of cyclin expression and the progressive inhibition of the Cdk1 inhibitor Swe1 (Wee1 in mammals). At the metaphase-anaphase transition, Cdk1 activity decreases sharply due to the combined action of the two APC/C complexes that degrade cyclins, along with the accumulation of the Cdk1 inhibitor Sic1 in budding yeast (p21 in mammals) (Morgan 2007; Sullivan and Morgan 2007) (Fig. 1). The number of cyclins and Cdk1s has increased during evolution, and mammals possess many more cyclins and Cdks than simpler organisms such as yeast. A long-standing question in the field is whether cell cycle progression is controlled primarily by the global level of Cdk1 activity or by the distinct substrate specificities of different cyclin-Cdk1 complexes.

In 2000, a major breakthrough came from the Shokat laboratory with the development of an analogue-sensitive Cdk1 allele (Cdk1-as or Cdk1 Shokat allele), in which the conserved gatekeeper residue was mutated (F88G) to enlarge the catalytic pocket. This modification preserves ATP binding while rendering Cdk1-as uniquely sensitive to bulky ATP analogues, such as 1-NM-PP1 or 3-MB-PP1, enabling rapid, dose-dependent, and reversible inhibition (Bishop et al. 2000). Crucially, it combines unique specificity with minimal off-target inhibition. In budding yeast, the first use of this allele allowed in vivo analysis of the cell cycle and the identification of around 180 Cdk1 substrates (Ubersax et al. 2003). Subsequent developments expanded the system beyond inhibition: chemically modified ATP analogues, such as N⁶-(benzyl)ATPγ-S or N⁶-(benzyl)γ-^32^P-ATP, allow selective labeling of Cdk1-as substrates, a strategy collectively termed ASKA (Analogue-Sensitive Kinase Allele), which enables direct identification of kinase targets (Blethrow et al. 2004, 2008; Allen et al. 2007). In 2009, David Morgan’s laboratory combined the Cdk1-as allele inhibitory strategy with global SILAC (Stable Isotope Labeling with Amino acids in Cell culture)-based quantitative mass spectrometry. Specific chemical inhibition of Cdk1 in vivo revealed 547 phosphorylation sites on 308 Cdk1 substrates. Strikingly, ~ 90% of sites were predicted to lie within loops or intrinsically disordered regions (IDRs). Moreover, Cdk1 sites tended to cluster, suggesting that multiple phosphorylations together modulate substrate surface properties. Evolutionary analysis across 32 fungal species showed that the precise position of phosphosites is not conserved, but clustering within IDRs is (Holt et al. 2009). These results suggested that a global phosphorylation environment—rather than single phosphorylation events—creates a “phosphocode” governing protein behavior. This concept of phosphorylation in IDRs was confirmed in 2023. IDRs naturally contain many serines, threonines, arginines, lysines, and prolines, so it was important to distinguish whether Cdk1 targets these regions because of their amino acid composition or because of “true” kinase preference. Using bioinformatics to correct for amino acid frequency biases, Valverde et al., showed in *Xenopus* embryos that Cdk1 phosphorylation is genuinely enriched in IDRs. Interestingly, Cdk1 substrates tend to be more intrinsically disordered than those of other proline-directed kinases like MAPKs (Mitogen-Activated Protein Kinases) (Valverde et al. 2023). A complementary study extended Holt’s idea of multisite clustering. By analyzing the transcription factor Hcm1, a Cdk1 substrate, they tested 256 combinations of eight SP/TP phosphosites. They found that multiple sites must be phosphorylated to produce a functional effect, and that different site combinations can lead to similar outcomes. Both the number and the arrangement of phosphorylations fine-tune protein behavior, showing that phosphorylation acts as an integrated network rather than a simple on/off switch (Conti et al. 2023). For a comprehensive overview of how CDK phosphorylation within IDR contributes to large-scale cellular remodeling and phase separation, see the recent review by Krasinska and Fisher (Krasinska and Fisher 2022).

Twenty years after its creation in yeast, the Cdk1-as system was adapted to mammals. Piotr Sicinski’s laboratory generated a Cdk1-as knock-in mouse to study how Cdk1 controls the epigenetic landscape in embryonic stem cells. Unlike in yeast, where the Cdk1-as allele alone supports viability, Cdk1-as/as homozygous mice die around embryonic day 10.5, suggesting that the analogue-sensitive kinase cannot robustly use natural ATP in vivo. Nevertheless, embryonic stem cells derived from these mice proliferate normally in the absence of inhibitor, while addition of the ATP analogue arrests cells in G2/M, mirroring observations in budding yeast (Michowski et al. 2020). Beyond inhibition, the ATPγ-S analogue is commonly used to directly label Cdk1 substrates. Thiophosphorylation, a non-natural post-translational modification, can be detected with thiophosphate ester-specific antibodies after PNBM (p-nitrobenzyl mesylate) alkylation (Hertz et al. 2010). Piotr Sicinski’s laboratory demonstrated this by showing that labeled Cdk1 substrates can be immunoprecipitated and identified by mass spectrometry, and the method is also compatible with immunoblotting and immunofluorescence. Together, these ASKA strategies—ranging from selective inhibition to thiophosphate labeling—provide a comprehensive toolkit to study Cdk1 activity, substrate specificity, and phosphorylation dynamics across yeast and mammalian systems, bridging mechanistic studies of kinase regulation with global substrate mapping (Michowski et al. 2020).

### Cdk1 quantitative and qualitative control of the cell cycle

Over the past 30 years, two models, quantitative and qualitative, have been proposed to explain cell-cycle regulation by addressing how the timing of substrate phosphorylation and dephosphorylation is controlled, thereby ensuring phase-specific processes such as DNA replication in S phase and chromosome segregation in M phase. These concepts were proved using the Cdk1-as system in *Saccharomyces cerevisiae* (budding yeast) and *Saccharomyces pombe* (fission yeast), with key contributions from the laboratories of Paul Nurse, Mart Loog, David Morgan, and Frank Uhlmann (Morgan 2007; Sullivan and Morgan 2007; Queralt and Uhlmann 2008; Uhlmann et al. 2011; Nurse and Hayles 2019; Valk et al. 2023). More recently, advances in high-resolution, time-resolved phosphoproteomics have allowed quantitative mapping of thousands of phosphorylation sites, revealing the dynamic interplay between kinases and phosphatases and providing a systems-level view of how quantitative and qualitative mechanisms are implemented across the phosphoproteome (Holt et al. 2009; Kuilman et al. 2015; McCloy et al. 2015; Cundell et al. 2016; Swaffer et al. 2016, 2018; Godfrey et al. 2017; Powers and Hall 2017; Baro et al. 2018; Touati et al. 2018, 2019; Touati and Uhlmann 2018; Jones et al. 2020; Pirincci Ercan et al. 2021; Basu et al. 2022; Curran et al. 2025; Zeisner et al. 2025; Takacs et al. 2025).

Note that in this review, I refer to this two historically but overlapping models for explaining temporal ordering of Cdk1 phosphorylation. In the quantitative model, ordering primarily emerges from gradual changes in the global level of Cdk1 activity and opposing phosphatases, such that substrates with different effective activation thresholds respond at different times. In the qualitative model, ordering is instead shaped by substrate-specific molecular features. These include cyclin docking interactions, motif optimality, priming, multisite organization, which tune how individual substrates interpret a given level of Cdk1 activity. Importantly, these two views are not mutually exclusive. Several mechanisms discussed in the “qualitative” framework (such as Cks1-dependent docking, serine-versus-threonine preferences, phosphomotif specificity) also influence the quantitative sensitivity of substrates to kinase activity. To make the review easier to follow, I discuss these mechanisms in the section on the qualitative model, while acknowledging that they also contribute to quantitative threshold behavior.

### Quantitative model: activity thresholds across cell cycle phases

#### In fission yeast

In 1996, Stern and Nurse first proposed that cell cycle progression is ordered by quantitative changes in Cdk1 activity, giving rise to the so-called quantitative model. According to this view, the progressive rise in Cdk1 activity through interphase, rather than cyclin identity, controls the timing of substrate phosphorylation and the order of events. Cdk1 activity is low during G1/S phase and rises first to a moderate threshold that triggers S phase entry, and later to a higher threshold that triggers G2/M entry. Once this threshold is reached, cells irreversibly enter mitosis, ensuring temporal separation of DNA replication and chromosome segregation. Substrate phosphorylation peaks in metaphase, after which Cdk1 activity is downregulated and substrates are progressively dephosphorylated, an essential step to reset the cycle for the next round (Stern and Nurse 1996).

The quantitative model was formally demonstrated in fission yeast in 2010 by Coudreuse and Nurse, who showed that the cell cycle can be controlled by a single cyclin-Cdk1 construct. They engineered a cassette expressing the only Cdk1 in fission yeast, cdc2, fused to the only B-type cyclin, cdc13, separated by a small linker (cdc13-L-cdc2). This construct was integrated into the genome under the cdc13 promoter, ensuring that both the catalytic subunit (cdc2) and the regulatory subunit (cdc13) were subject to the same transcriptional, translational, degradation, and localization programs. The fusion also prevented binding to alternative partners. After deleting the endogenous copies of cdc2 and cdc13 (Δ2 Δ13), the cell cycle functioned normally. Even when the three other major cyclins clustering with cdc13 named cig1, cig2, and puc1 were deleted (ΔCCP), a single cyclin-Cdk1 construct (cdc13-L-cdc2 Δ2 Δ13 ΔCCP) was still able to maintain cell cycle progression. To demonstrate that different levels of Cdk1 activity control S and M phases, they replaced the cdc2 allele with the Shokat analogue-sensitive version (cdc13-L-cdc2-as Δ2 Δ13 ΔCCP) and modulated Cdk1 activity dose-dependently using the ATP analogue 1-NM-PP1. Different inhibitor concentrations produced distinct responses: 500 nM halted the cell cycle in G2/M, whereas 5 µM blocked it in both G1 and G2/M. These results support the view that G1/S and G2/M transitions are governed by distinct Cdk1 activity thresholds: lower for S phase and higher for M phase (Coudreuse and Nurse 2011). This single cyclin-Cdk1 strain has since been used for mathematical modeling to investigate why loss of Cdk1 inhibitory phosphorylations by Wee1 and Myt1 kinases is tolerated in this context but not in wild-type cells (Gérard et al. 2015), and to study replication origin selection patterns (Perrot et al. 2018).

In 2016, after decades of genetic and biochemical studies focused on kinases and cyclins, it was finally possible to directly observe, on a global scale, how their substrates are modified with time. Swaffer et al. extended our current understanding by examining how a single source of cyclin-Cdk1 activity can regulate the cycle using time-resolved mass spectrometry. Comparing systems with either multiple cyclins (cdc13-L-cdc2-as) or a single cyclin (cdc13-L-cdc2-as Δ2 Δ13 ΔCCP), they found that distinct sets of Cdk1 substrates were phosphorylated at the expected times: DNA replication substrates in S phase and mitotic substrates in M phase. M-phase Cdk1 activity was approximately ten-fold higher than in S phase, with early substrates more sensitive to low activity than late substrates. Releasing G1-arrested cells into high Cdk1 activity caused mitotic catastrophe, with S- and M-phase phosphorylation events overlapping. These findings provide large-scale evidence that substrate phosphorylation order is controlled by Cdk1 activity thresholds, independently of cyclin specificity (Swaffer et al. 2016). Although Cdc13-Cdc2 localizes mainly to the nucleus, it concentrates at the spindle pole body (SPB, the yeast equivalent of the centrosome) in late G2 and on the spindle during mitosis. This distribution may influence substrate access, but it cannot fully explain why early substrates are phosphorylated more efficiently.

In 2020, Basu et al., further refined this view, showing that the hydrophobic patch on Cdc13 (M-phase cyclin) is essential for SPB localization, a function conserved in human cyclin B1. Mutation of this patch selectively reduced phosphorylation of half of the mitotic substrates, particularly those requiring the highest Cdk1 activity and caused G2 arrest without affecting S phase. Subsequent studies using dual Cdk1 activity sensors (NucCdk1 for nuclear Cdk1 and CytCdk1 for cytoplasmic Cdk1) demonstrated that nuclear Cdk1 activation precedes cytoplasmic activation (Kapadia and Nurse 2025). These findings indicate that while Cdc13 SPB localization is not required for nuclear Cdk1 activity, it is essential for Cdc13-Cdc2 translocation to the cytoplasm and subsequent phosphorylation of SPB and cytoplasmic targets. Together, these results highlight how Cdc13 SPB localization enforces the sequential order of nuclear and cytoplasmic Cdk1 activation, ensuring proper timing of mitotic events (Basu et al. 2020). Finally, in 2022, Basu et al., examined whether the S-phase cyclin Cig2 could compensate for loss of the M-phase cyclin Cdc13. By varying Cig2-Cdk1 (S-Cdk1) and Cdc13-Cdk1 (M-Cdk1) levels, they found that 13% of M-Cdk1 substrates could not be phosphorylated by S-Cdk1, while 22% of S-Cdk1 substrates were not phosphorylated by M-Cdk1. Thus, S-Cdk1 phosphorylates a broader subset of substrates than M-Cdk1. Remarkably, 65% of substrates were phosphorylated equally by both challenging a strict qualitative or quantitative model. Importantly, removal of the phosphatase PP1 from the SPB enabled S-Cdk1 to phosphorylate additional M-Cdk1 substrates. Under these conditions, S-Cdk1 alone was sufficient to drive mitotic entry and progression in the absence of M-Cdk1 (Basu et al. 2022). These results underscore the central role of phosphatases in enforcing activity thresholds and preventing overlap between cell cycle phases.

#### In budding yeast

In budding yeast, Pirincci Ercan et al. attempted to engineer a single-cyclin strain. They showed that Clb2, the major M-phase cyclin, can substitute for other B-type cyclins (Clb1, Clb3, Clb4). Clb2 can also replace S-phase cyclins (Clb5, Clb6) when expressed from both Clb2 and Clb5 promoters (Cln-Clb2^S−M^ strain). However, S-phase timing was delayed, and global mass spectrometry analysis showed that mitotic events became less orderly, showing partial but not complete compensation. The authors also attempted to eliminate G1 cyclins (Cln1-3). They succeeded in deleting Cln1 and Cln3, generating a “Cln2-Clb2^S−M^” strain, but could not delete Cln2 under any tested condition (even when Sic1 and Swe1 were deleted, or with Clb2 expressed from the Cln2 promoter). Therefore, they developed a conditional depletion system (MET3prCln2-Clb2^G1−S−M^). Upon Cln2 repression, cells failed to bud, and half showed delayed DNA replication and spindle elongation. This demonstrated that Cln2 is essential for polarity control and substrates involved in budding pathway, roles that Clb2 cannot assume (Pirincci Ercan et al. 2021). These findings suggest that budding yeast, whose division depends on budding, relies more heavily on cyclin specificity than fission yeast. Together, these results indicate that budding yeast does not exhibit the same cell cycle plasticity as fission yeast, and that a purely quantitative model of Cdk1 activity is insufficient to drive orderly cell cycle progression.

Importantly, the proper ordering of substrate dephosphorylation during mitotic exit relies on the quantity of opposing phosphatases, in combination with the decrease of Cdk1 activity. The major phosphatase counteracting Cdk1 during mitotic exit is Cdc14. Its activity rises progressively during mitotic exit through a two-step release: first from the nucleolus to the nucleus in early anaphase via the Cdc14 Early Anaphase Release (FEAR) network, and later from the nucleus to the cytoplasm via the Mitotic Exit Network (MEN) (Visintin et al. 1998). In 2011, Bouchoux & Uhlmann showed that the balance between declining Cdk1 activity (through B-type cyclin degradation and Sic1 accumulation) and rising Cdc14 activity ensures ordered substrate dephosphorylation. In this context, the best Cdc14 targets or weakest Cdk1 substrates are dephosphorylated first, referred to as early substrates, while poor Cdc14 targets or strong Cdk1 substrates are dephosphorylated last, referred to as late substrates. This temporal ordering ensures that substrates controlling spindle elongation (early substrates) are reset before those controlling cytokinesis (late substrates), preserving the proper sequence of mitotic exit events (Bouchoux and Uhlmann 2011). We extended these findings with in vivo data. Large-scale phosphoproteomics revealed that Cdc14 dictates temporal order by preferentially targeting serine-containing Cdk1 motifs before threonine motifs. In the Cdc14 auxin-inducible degron mutant, this order is reversed, causing the different waves of substrate dephosphorylation to collapse, leading to cytokinesis defects and the formation of chains of interconnected cells. In contrast, mutants for PP2A^Cdc55/B55^ or PP2A^Rts1/B56^ did not display these defects (Touati et al. 2019). These results confirm Cdc14 as the master phosphatase in budding yeast, orchestrating ordered dephosphorylation during mitotic exit, thus extending the quantitative model to incorporate both kinase and phosphatase activities also in budding yeast. Although the quantitative model emphasizes global changes in Cdk1 and phosphatase activity, several layers of substrate-specific features further refine these thresholds, a point developed in the qualitative model below.

### Qualitative model: cyclin specificity and substrate recognition

**Fig. 2:**
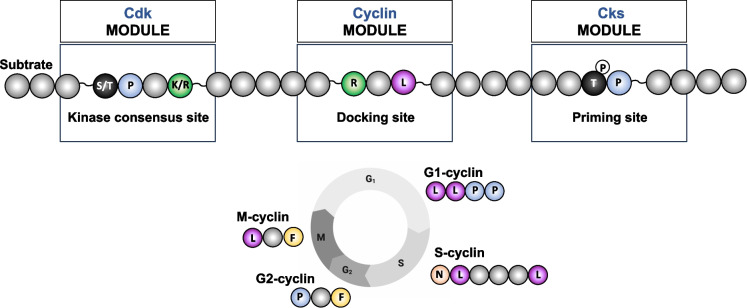
Multiple layers of phosphocode recognition determine the timing of substrate phosphorylation by cyclin-Cdk-Cks complexes. Schematic representation of the combinatorial recognition code governing Cdk substrate phosphorylation, as described by the Loog laboratory. Cyclin-Cdk-Cks complexes recognize substrates through multiple modules: the phosphomotif, short linear docking motifs (SLiMs) specific to each cyclin, and priming site promoting processive phosphorylation. The sequential expression of cyclins during the cell cycle allows Cdk1 to engage distinct docking interactions, with the most characterized SLiMs indicated for each cyclin. The spacing between modules is critical. Processivity typically occurs when two motifs are separated by approximately 10–22 amino acids in disordered regions. In addition, suboptimal or minimal motifs such as (S/T)P or (S/T)xx(K/R) can be efficiently phosphorylated within these modular frameworks, enabling efficient multisite phosphorylation and tight temporal regulation

The first definition of the qualitative model suggests that the timing of substrate phosphorylation and dephosphorylation is influenced by the temporal expression of cyclins. Distinct cyclin-Cdk1 complexes use specific strategies to recognize their substrates. Some sites on cyclins recognize SLiMs (Short Linear Motifs) located at a distance from the phosphorylation site on substrates. These docking motifs act as kinase binding sites and influence phosphorylation kinetics. While this was the initial explanation for the qualitative model, major advances over the last decade, particularly from Mart Loog’s laboratory using synthetic biology, have revealed that substrate recognition involves a combination of features at specific distance, not only docking motifs (Fig. 2) (Ord and Loog 2019; Valk et al. 2023).

Indeed, Cdk1 kinase activity is unique in that it does not act as a simple ON/OFF switch. Instead, it operates through multiple activity thresholds and regulatory layers that fine-tune substrate phosphorylation. These thresholds are interpreted differently by each substrate depending on their molecular features, so not all targets respond to the same level of Cdk1 activity. Targets are recognized by kinases and phosphatases via clustered multiphosphorylation and docking sites, forming a multi-layered code on substrate surfaces. For simplicity, I classified all the molecular features that define this code under the qualitative model of Cdk1 control. These features include amino acid type, flanking phosphomotifs, docking regions, priming phosphorylations, and accessibility in ordered versus disordered regions. This combinatorial code ultimately determines whether, when, and how efficiently a substrate is phosphorylated or dephosphorylated in response to the same Cdk1 activity (Holt et al. 2009; Salazar and Höfer 2009; Roy and Cyert 2009; Godfrey et al. 2017; Nilsson 2019; Hoermann and Köhn 2021; Kokot and Kohn 2022; Conti et al. 2023; Valverde et al. 2023).

#### Phosphoresidues and phosphomotifs

Cdk1 activity dominates cell cycle progression, but the oscillating phosphorylation landscape also reflects inputs from numerous other kinases. Each kinase recognizes specific consensus motifs and shows selectivity for the phosphoacceptor (serine versus threonine), determined by the residue immediately following the canonical DFG motif in the activation loop: F/Y/W favors serine, while V/I/T favors threonine (Johnson et al. 2023). Cdk1 recognizes a “minimal” consensus motif defined by a serine or threonine (phosphoresidue) followed by a proline (S/T)P (phosphomotif). However, the efficiency of phosphorylation strongly depends on the local sequence context. Residues located at + 1 and + 3 relative to the phosphoacceptor site refine this minimal consensus into an optimal or “full” Cdk motif, typically represented as (S/T)Px(K/R). Basic residues at position + 3 enhance catalytic efficiency, whereas bulky or hydrophobic residues tend to reduce it. In addition, serines are generally phosphorylated more efficiently than threonines, contributing to the temporal ordering of substrate modification. Together, these local preferences generate a spectrum of phosphorylation kinetics, where optimal motifs respond to low Cdk1 activity, while suboptimal motifs require higher thresholds (Ubersax et al. 2003; Holt et al. 2009; Errico et al. 2010; Suzuki et al. 2015; Michowski et al. 2020).

In reality, the Cdk1 phosphorylation landscape is far more diverse than these textbook consensus motifs. In vitro peptide array and phosphoproteomic studies in yeast and mammals revealed numerous unconventional Cdk1 sites. Using in vitro kinase assays with an arginine-scanning peptide library, Suzuki et al. (2015) compared cyclinB1-Cdk1 and ERK2 (a MAP kinase). CyclinB1-Cdk1 phosphorylated a broader range of sequences, including non-classical sites, whereas ERK2 was strongly restricted to canonical SP/TP motifs. Non-conventional Cdk1 sites lacking proline at + 1 ((S/T)xx(K/R)) were still phosphorylated efficiently. Importantly, the presence of either + 1P or + 3(K/R) remained essential (Suzuki et al. 2015). These findings have been confirmed in vivo. In budding yeast, Cln1/2-Cdk1 complexes target Px(S/T)P(K/R) motifs, with a basic residue at + 2 rather than + 3, and phosphorylation is enhanced by a proline at − 2 (Kõivomägi et al. 2011). In mammals, Cdk1 phosphorylation is promoted by lysines at + 2 to + 5, and cyclin A2-Cdk1 can target many (S/T)xx(K/R) sites lacking + 1P (Michowski et al. 2020; Al-Rawi et al. 2023).

We showed by time-resolved phosphoproteomics that each phosphosite, rather than each substrate, is subject to its own regulation. Clearly, different Cdk1 consensus motifs (full, minimal, conventional, unconventional) have their phosphorylation peaking at different times during the cell cycle. For example, serine residues on full Cdk1 consensus sites (SPx(K/R)) are phosphorylated and dephosphorylated earlier than threonine residues on TPx(K/R) sites, highlighting the importance of the phosphosignature for proper timing of substrate phosphorylation during the cell cycle (Godfrey et al. 2017; Touati et al. 2019). A clear example shows that non-conventional sites and negative selection are equally important as conventional sites to prevent cross-phosphorylation, while the absence of such restrictions allows overlap between pathways (Johnson et al. 2023). Non-conventional sites regulate nuclear localization and export signals, but also protein degradation through phosphodegrons (Kõivomägi et al. 2011, 2012; Faustova et al. 2021a; Valk et al. 2023). Thus, non-conventional motifs expand Cdk’s functional repertoire. They allow selective phosphorylation by Cdks while avoiding phosphorylation by other proline-directed kinases such as MAPKs, and at the same time, they provide a range of phosphorylation rates and thresholds.

The subtlety of conventional and non-conventional consensus motifs has been less studied for other cell cycle kinases. Among major cell cycle kinases, Polo-like kinase (Cdc5^Plk1^) and Aurora kinase (Ipl1) are essential. Cdc5^Plk1^ is required for mitotic entry, chromosome segregation, and cytokinesis (Botchkarev and Haber 2017), whereas Ipl1^Aurora^ ensures chromosome biorientation and segregation and contributes to cytokinesis in higher eukaryotes (Afonso et al. 2017). Their consensus motifs are (D/E/N)x(S/T) for Plk1^Cdc5^ and (K/R)(K/R)x(S/T) for Aurora^Ipl1^ (Mok et al. 2010; Alexander et al. 2011). Other budding yeast mitotic kinases include the MEN kinases Cdc15 and Mob1-Dbf2, and the RAM (Regulation of Ace2 and Morphogenesis) pathway kinase Mob2-Cbk1, which preferentially phosphorylate RxxS motifs (Weiss 2012). Our time-resolved global phosphoproteomic data also show that specific kinase consensus motifs are phosphorylated at distinct cell cycle stages, with Cdc5^Plk1^ consensus motifs peaking before Ipl1^Aurora^ motifs and the other MEN and RAM kinase pathways (Touati et al. 2018). Similar results have been observed in fission yeast, emphasizing that the composition of phosphomotifs modulates phosphorylation kinetics (Swaffer et al. 2018).

Several phosphatases coordinate the timing of Cdk1 substrate phosphorylation during cell cycle progression. Once thought to be promiscuous, their phosphomotif specificity has recently been deeply studied by time-resolved mass spectrometry in budding and fission yeast. In budding yeast, Cdc14 is the main phosphatase counteracting Cdk1 activity during the metaphase-to-anaphase transition. Cdc14 preferentially dephosphorylates serine-based SPx(K/R) motifs (Bremmer et al. 2012). Using a Cdc14 degron system, we have shown that these motifs are particularly affected by Cdc14 loss, perturbing the first wave of substrate dephosphorylation (Touati et al. 2019).

In addition to Cdc14, PP2A plays a major role during mitosis. In budding yeast, PP2A is a trimeric complex composed of a scaffolding subunit (Tpd3), a catalytic subunit (Pph21 or Pph22), and one of three regulatory subunits Cdc55 (B55 in mammals), Rts1 (B56), or Rts3 (Moyano-Rodriguez and Queralt 2019). Choice of the regulatory subunit strongly influences the catalytic preferences of the holoenzyme. Strikingly, we have shown that PP2A^Cdc55/B55^, but not PP2A^Rts1/B56^, preferentially dephosphorylates threonine residues within Cdk1 motifs. This threonine bias delays the phosphorylation of specific substrates during interphase, such as Sli15 (Aurora activator) and Ndd1 (Clb2 transcription factor), thereby modulating the temporal order of mitotic entry (Godfrey et al. 2017). Moreover, deletion of either Cdc55^B55^ or Rts1^B56^ regulatory subunit affects the dephosphorylation of different phosphomotifs during mitotic exit. Thus, phosphatases operate via two complementary strategies, delaying phosphorylation of selected residues during interphase and accelerating dephosphorylation at mitotic exit. A similar partitioning of Cdk1 substrate classes among phosphatases has also been observed in fission yeast, where distinct phosphatases, including PP2A^B55^, PP2A^B56^, Cdc14, and PP1, each target specific subsets of phosphomotifs (Zeisner et al. 2025). Phosphatases, as kinases, have phosphomotif preferences, and this concept also holds true and is conserved in mammals (Hein et al. 2017; Kruse et al. 2020; Hoermann et al. 2020). Moreover, the regulatory subunits of PP2A do more than target the phosphatase to its substrates. Indeed, they reshape the catalytic preferences of the enzyme itself, thereby modifying the first-level phosphocode that dictates phosphorylation dynamics throughout the cell cycle.

#### Docking motifs

**Fig. 3:**
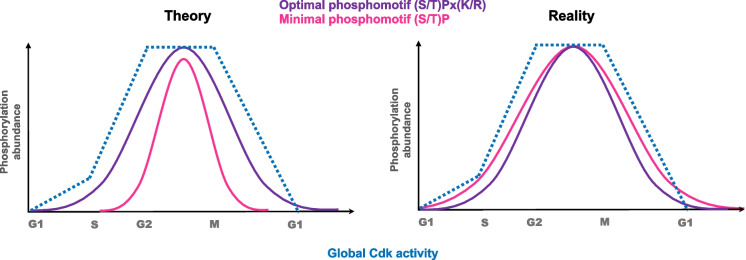
Theoretical versus actual behavior of optimal and suboptimal Cdk1 phosphorylation sites. In a minimal network model (left) considering only kinase motif specificity (the first layer of the phosphocode) optimal motifs such as (S/T)Px(K/R) (purple) are predicted to be efficiently phosphorylated across most of the cell cycle, following quantitative changes in global Cdk1 activity. In contrast, suboptimal motifs such as (S/T)P (pink) would be expected to display a narrower phosphorylation window. In reality (right), the integration of multiple layers of the phosphocode, including docking interactions, priming events, and phosphatase counteraction, allows even minimal motifs to become phosphorylated over a broader temporal range, even spanning several cell cycle phases. Figures and concepts adapted from Paul Nurse, Mart Loog labs and our phosphoproteomic results

Conventional and non-conventional kinase consensus motifs help prevent kinases from targeting each other but they often share similar consensus motif specificities (Suzuki et al. 2015). Moreover, concerning Cdk1 phosphomotifs, many suboptimal/unconventional Cdk1 sites are phosphorylated early in G1/S, prompting the question of how they achieve full phosphorylation stoichiometry so rapidly (Swaffer et al. 2016, 2018; Godfrey et al. 2017; Touati et al. 2018) (Fig. 3). These observations indicate that other layers of the code are required to achieve both signaling specificity and efficient phosphorylation of suboptimal Cdk1 sites.

Docking motifs provide an additional level of discrimination between substrates and compensate for suboptimal Cdk1 consensus motifs. Over the last decade, our understanding of docking-based strategies in budding yeast has expanded massively. Contrary to the previously established dogma, cyclins are not limited to a single docking strategy and can recognize multiple motifs. Many cyclins can engage the same docking motif, particularly RxL motifs, providing flexibility in substrate recognition. Surprisingly, even Clb2-Cdk1, the most potent M-phase cyclin, relies on a specific docking site for certain substrates. (Ord et al. 2019b). Briefly, G1, S, G2, and M cyclins (Cln2, Clb5, Clb3, and Clb2, respectively) recognize distinct “main” short linear docking motifs—LLPP, NLxxxL, PxF, and LxF—that confer substrate specificity (Fig. 2). As mentioned, recognition of the RxL motif is shared among several cyclins with different affinity (Bhaduri and Pryciak 2011; Bhaduri et al. 2015; Ord et al. 2019a, b, 2020; Venta et al. 2020; Bandyopadhyay et al. 2020; Faustova et al. 2021a, b, 2022; Koivomagi et al. 2021). These recognition motifs locally enrich kinase concentration near substrates and can even target the kinase to specific cellular structures, such as the SPB, increasing phosphorylation efficiency of localized substrates (Basu et al. 2020). The strength of docking modulates the extent and timing of phosphorylation, in a manner analogous to the effect of consensus motifs (Ord et al. 2020; Bandyopadhyay et al. 2020). Also, recent evidence from yeast and mammalian systems indicates that cyclin docking is not restricted to SLiMs and can additionally be mediated by amphipathic helices (Topacio et al. 2019; Faustova et al. 2021b).

This logic extends to phosphatases, which also use docking motifs to achieve substrate specificity. Cdc14 preferentially binds substrates containing a PxL docking motif (Kataria et al. 2018). The presence of a PxL motif on the Csa1 protein, an SPB anchor for Cdc5^Plk1^, strongly influences its phosphorylation status, suggesting that phosphatase docking motifs can locally modulate the Cdk1 activity threshold (Ord et al. 2020). Similarly, PP2A holoenzymes use distinct docking strategies: PP2A-B56 binds LxxIxE motifs through its regulatory subunit pocket, while PP2A-B55 targets substrates with polybasic sequences flanking the phosphosite (Cundell et al. 2016; Hertz et al. 2016; Wang et al. 2016). Thus, both kinases and phosphatases use combinatorial docking strategies that define the multiphosphosite code, coordinating the spatial and temporal control of substrate phosphorylation.

#### Priming phosphorylation

While consensus motifs and docking sites offer multiple levels of substrate recognition, they cannot fully account for the complexity of Cdk1-dependent signaling. Hence, another level of regulation emerges through priming phosphorylation, which integrates information from existing phosphorylation marks to modulate subsequent events. This priming can occur through Cdk1 itself (intramolecular priming) or through other kinases, establishing hierarchical phosphorylation cascades. Such priming events promote processive phosphorylation of neighboring sites, turning graded kinase activity into switch-like substrate responses (Thomson and Gunawardena 2009).

The best-characterized example comes from the Cdk1 cofactor Cks1, which specifically recognizes phosphorylated TP motifs and anchors cyclin-Cdk1 complexes onto their substrates (Kõivomägi et al. 2011; McGrath et al. 2013; Valk et al. 2023). This docking promotes efficient phosphorylation of neighboring suboptimal sites, enabling processive multisite phosphorylation once the kinase is engaged. The effect is highly distance-dependent: the priming site typically lies within 10–22 amino acids of the next target site in IDRs (Kõivomägi et al. 2013). Importantly, Cks1-mediated priming is directional: the primed phosphosite is positioned N-terminal to downstream target sites, imposing an N-to-C vectorial phosphorylation that constrains how multisite clusters are decoded. Such multisite clusters, frequently located in IDRs, act as information-processing modules. Once Cdk1 is docked through Cks1, multiple nearby residues can be phosphorylated without dissociation, producing graded and temporally ordered phosphorylation responses (Fig. 2). This mechanism generates distinct activity thresholds, allowing substrates to respond quantitatively to progressive increases in Cdk1 activity. Work from Mart Loog’s group elegantly demonstrated that the positioning of priming and docking motifs modulates these thresholds and drives diverse functional outputs, such as phosphodegron formation or nucleocytoplasmic transport (Faustova et al. 2022). In 2025, to dissect the global contribution of Cks to Cdk-dependent phosphorylation, Curran et al., combined thermosensitive Cks mutants (suc1-ts) with quantitative phosphoproteomics in fission yeast. Cells were synchronized in G1 and G2, and phosphosite dynamics were compared between wild type and Cks mutants. This approach identified 221 phosphorylation sites on 133 proteins whose modification depended on Cks. Motif enrichment analysis revealed that Cks-dependent sites were markedly depleted in the canonical Cdk consensus (+ 1 proline), showing that Cks enables phosphorylation of suboptimal or non-canonical motifs that would otherwise remain unmodified (Curran et al. 2025).

A similar principle applies to other kinases. The Polo-box domain (PBD) of Plk1 represents another striking example of priming phosphorylation strategy. The PBD selectively recognizes pre-phosphorylated sites on substrates, allowing Plk1 to dock and phosphorylate additional residues in a controlled and processive manner. This priming-dependent docking is conceptually analogous to the Cks1-Cdk1 mechanism, as it depends on the phosphorylation state of the substrate rather than on its phosphomotif or a canonical docking motif (Elia et al. 2003; Lee et al. 2014). Together, these examples show that priming phosphorylation adds an additional layer to the phosphocode. By using existing phosphorylation marks to guide new ones, priming mechanisms allow kinases to read and build complex phosphorylation patterns, ensuring accurate timing and localization of signaling events.

### Reconciliation around a hybrid model

Kinase and phosphatase abundance and activity vary throughout the cell cycle, establishing quantitative thresholds that determine when substrates are phosphorylated or dephosphorylated. In parallel, substrate-encoded molecular features act qualitatively to determine kinase-substrate recognition. As mentioned, qualitative and quantitative mechanisms are not mutually exclusive. Molecular features grouped under a qualitative model modulate how substrates respond to increasing Cdk1 activity and therefore act in concert with quantitative threshold mechanisms. Paul Nurse’s laboratory introduced the concept of a hybrid model (Basu 2022). In parallel, Mart Loog’s group proposed a “unified model of Cdk1 function,” emphasizing that quantitative and qualitative mechanisms should be viewed as complementary rather than opposing (Ord and Loog 2019). A first piece of evidence supporting a hybrid model emerged in 2005, when Loog and Morgan performed a screen using purified analog-sensitive Cdk1 bound to either Clb2 or Clb5 to distinguish M-phase (Clb2-Cdk1) from S-phase (Clb5-Cdk1) substrates. They identified fourteen Clb5–Cdk1–specific targets whose recognition depends strongly on RxL-mediated docking to the cyclin hydrophobic patch. No Clb2-specific substrates were found, but the authors proposed that cyclin binding not only activates Cdk1 but also modulates the intrinsic catalytic activity of each cyclin-Cdk1 complex, meaning its inherent ability to phosphorylate substrates is independent of docking interactions (Loog and Morgan 2005). Subsequent kinetic analyses by Loog’s laboratory compared four cyclin-Cdk1 modules—Cln2-Cdk1, Clb5-Cdk1, Clb3-Cdk1, and Clb2-Cdk1—using a generic histone H1 peptide bearing the optimal Cdk1 motif but lacking docking elements (Fig. 1). They observed a gradual increase in intrinsic catalytic efficiency from early to late cyclins, suggesting that docking interactions compensate the weaker activity of early complexes. This progressive increase in activity ensures sequential cell-cycle transitions and prevents the collapse of successive phases. The inhibitory kinase Swe1 further modulates the most active complexes, such as Clb2-Cdk1, providing early evidence that regulatory subunits can fine-tune the intrinsic catalytic power of Cdks (Kõivomägi et al. 2011).

Extending these findings to a global scale, quantitative proteomics from the Nurse’s laboratory confirmed that S-cyclin-Cdk1 and M-cylin-Cdk1 phosphorylate most substrates with similar efficiency, with only a minority displaying clear cyclin preferences. Importantly, this functional specialization could be bypassed by increasing Cdk activity or reducing counteracting phosphatases, such as PP1, emphasizing that cell-cycle control largely follows a quantitative logic with local modulatory layers (Basu 2022; Basu et al. 2022). Building on this quantitative view, the hybrid model extends Cdk regulation by integrating the action of counteracting phosphatases. These enzymes do not simply erase kinase activity but actively shape its temporal readout, converting gradual changes in Cdk1 activity into ordered dephosphorylation waves. Among them, Cdc14 in budding yeast plays a central role during mitotic exit, where its progressive activation follows the progressive decline of Cdk1 activity and ensures the sequential resetting of phosphorylated substrates. Through this antagonistic balance, phosphatases help enforce the correct order of substrate dephosphorylation, creating a robust temporal program that guides mitotic exit (Bouchoux and Uhlmann 2011; Uhlmann et al. 2011; Touati et al. 2019).

Phosphomotifs and docking motifs, while primarily defining substrate specificity, also integrate quantitative information about Cdk1 activity**.** Quantitatively, motifs with different optimality respond to distinct Cdk1 thresholds, ensuring that high-affinity sites are phosphorylated at lower kinase activity while suboptimal sites require stronger activity. Qualitatively, docking motifs guide cyclin-Cdk complexes to specific substrates, determining which proteins are modified. Other players such as Cks, traditionally viewed as passive phospho-adaptors linking Cdk to pre-phosphorylated TP motifs, have emerged as active modulators of both the quantitative and qualitative layers of Cdk regulation. Quantitatively, Cks lowers the effective Cdk activity required to phosphorylate weak motifs, while qualitatively, it influences substrate selection by enabling phosphorylation of atypical residues. By converting gradual increases in global Cdk activity into discrete, ordered phosphorylation waves, Cks reinforces the central logic of the hybrid model, coordinating the timing and specificity of substrate phosphorylation (Al-Rawi et al. 2023; Curran et al. 2025).

**Fig. 4:**
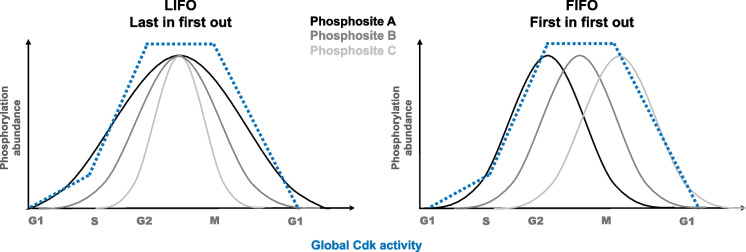
Comparison between the Last In First Out (LIFO) and First In First Out (FIFO) models of Cdk substrate phosphorylation. In the LIFO (Last In, First Out) model, less favorable phosphorylation sites accumulate more slowly and are dephosphorylated earlier, leading to a situation where the last sites to be phosphorylated are the first to be dephosphorylated. In contrast, the FIFO (First In, First Out) model reflects cases where phosphorylation and dephosphorylation follow the same sequential order in which sites are encountered. These models illustrate different temporal logics of substrate regulation, highlighting how phosphorylation order can be tuned to achieve precise control during the cell cycle**.** Figures and concepts adapted from Mart Loog lab

The diversity in substrate phosphorylation behavior reflects how quantitative thresholds and qualitative features interconnect. Some substrates require brief phosphorylation at specific times to trigger defined events, while others act as “safety locks,” remaining phosphorylated for extended periods, for instance, to prevent relicensing of DNA replication origins. Mart Loog’s laboratory formalized this logic through LIFO (Last In, First Out) and FIFO (First In, First Out) models. Here, LIFO corresponds to what is also called FILO (First In, Last Out); both terms describe the same kinetic logic. In the FILO (First In, Last Out) model, optimal phosphorylation sites are phosphorylated early and remain phosphorylated longer, whereas in the LIFO (Last In, First Out) model, less favorable sites are phosphorylated more slowly and are dephosphorylated earlier (Fig. 4). These dynamics emerge from the combined effects of docking interactions, priming phosphorylations, local kinase context, and phosphatase regulation. They integrate qualitative and quantitative influences to define the timing of phosphorylation and dephosphorylation. In contrast, the classical FIFO (First In, First Out) model describes substrates that are phosphorylated and dephosphorylated in the order they are encountered, for example on proteins where all sites are equally accessible. In this case, the timing of phosphorylation primarily reflects the overall kinase activity and site availability, with minimal contribution of substrate features (Fig. 4) (Kõivomägi et al. 2011; Ord et al. 2019b; Faustova et al. 2021a).

## Meiotic Cdk1 control

Having established the principles of Cdk1-dependent phosphorylation in mitosis, including the integration of qualitative and quantitative cues and the hybrid logic, we can now ask how these mechanisms are adapted during meiosis. Meiosis is a specialized division that halves the chromosome number, producing four genetically distinct haploid cells from a single diploid cell, and is essential for sexual reproduction. Unlike mitosis, meiosis consists of two consecutive M phases without an intervening S phase. The program begins with a prolonged pre-meiotic S phase, during which DNA replication occurs alongside homologous recombination and chromosome pairing. Homologous chromosomes segregate during the first meiotic division (MI), while sister chromatids segregate during the second (MII). Although MII closely resembles mitosis regarding chromosome attachment and orientation, MI is unique: homologs rather than sister chromatids segregate, requiring specialized mechanisms to ensure correct bi-orientation of homologs and co-orientation of sister chromatids, and an adapted spindle assembly checkpoint (SAC) to accommodate this configuration. A key challenge in meiosis lies in the MI-MII transition. In contrast to mitosis, S and M phases, normally tightly coupled in mitosis, must be uncoupled to allow two consecutive divisions without DNA re-replication. SPB duplication and spindle assembly must be reactivated for MII, while DNA replication is strictly prevented. To achieve this, only certain substrates must be reset by dephosphorylation to exit meiosis I, whereas others must remain phosphorylated to prevent DNA replication. Therefore, both the quantitative and qualitative models need to be revised to explain how cells handle this particular configuration in meiosis (Petronczki et al. 2003; Marston 2014; Touati and Wassmann 2016).

### Stage-specific cyclin-Cdk1 orchestration

In both yeast and mammals, certain cyclin-Cdk1 complexes are meiosis-specific, exhibiting distinct temporal activity patterns between meiosis I (MI) and meiosis II (MII). In budding yeast, the G1 cyclins Cln1-3 are not expressed during meiosis, while five of six M-phase cyclins are produced. Notably, the major mitotic cyclin Clb2 is transcriptionally repressed. Clb5-Cdk1 drives pre-meiotic S phase, whereas M-cyclins (Clb1, Clb3, Clb4) are initially absent to prevent mitotic-like events. Expression of M-cyclins is controlled by the transcription factor Ndt80, which activates gene expression in prophase I at pachytene (MacKenzie and Lacefield 2020; Palacios-Blanco and Martín-Castellanos 2022). Detailed analyses of Clb-Cdk1 complexes reveal precise temporal regulation. Clb5 accumulates earliest, with protein and kinase activity peaking in prophase I and early metaphase II. Clb4 remains stable from metaphase I to II, showing only a moderate drop in activity during anaphase I. Clb1 peaks from metaphase I to anaphase II, with a phospho-shift indicating activity largely restricted to meiosis I. Clb3 accumulates mRNA alongside other cyclins but is active only during meiosis II (Fig. 1). Misexpression of Clb3 in meiosis I leads to premature sister chromatid separation, underscoring the importance of strict temporal control (Carlile and Amon 2008). These observations suggest that some substrates require targeting by specific cyclins exclusively in meiosis II, likely employing docking strategies dependent on Clb3, possibly involving the PxF motif recognition observed in mitosis.

Attempts to support meiotic progression with a single cyclin have been far less successful than in mitosis. In budding yeast, unlike mitosis where M-cyclins display considerable redundancy and even a single Clb can drive the cell cycle, meiosis requires specialized, non-compensable roles for M-cyclins (Pirincci Ercan et al. 2021). Indeed, deletion of Clb1 alone is sufficient to impair sporulation, and combined deletions of Clb1 with Clb3 or Clb4 completely block meiotic divisions (Dahmann and Futcher 1995). A similar approach was attempted in fission yeast by Gutiérrez-Escribano and Nurse in 2014, inspired by Coudreuse and Nurse’s mitotic “single cyclin” strategy. They engineered a strain in which cdc2 (Cdk1) activity was driven exclusively by the M-phase cyclin cdc13 (the cdc13-L-cdc2 module), deleting all other interphase, mitotic, and meiotic cyclins. The cdc13-L-cdc2 Δ2 Δ13 ΔCCP strain (lacking endogenous cdc2, cdc13, and mitotic cyclins) was able to complete pre-meiotic S phase but arrested in meiosis I and formed 2-spores, failing to finish the second meiotic division. This defect could be rescued by providing four additional copies of the cdc13-L-cdc2 module, likely increasing protein abundance and overall Cdk1 activity, allowing most cells to form 3–4 spores. Deletion of the two meiotic-specific cyclins rem1 and crs1 (ΔRC) in this background (4 × cdc13-L-cdc2 Δ2 Δ13 ΔCCP ΔRC) produced spore numbers similar to wild type, but the two meiotic divisions succeeded only under azygotic conditions (h +/h +) and not in the natural zygotic context (h +/h-), where spore viability was strongly reduced and many cells formed more than four spores. These results illustrate that driving two M phases without an intermediate S phase using a single cyclin-Cdk1 source is substantially more challenging than driving the canonical alternation of S and M phases in mitosis (Gutierrez-Escribano and Nurse 2015).

The Cdk1-as allele has been used in meiosis to probe the quantitative control of progression, similar to mitosis. In Cdk1-as strains, addition of 0.5 µM 1-NM-PP1 in G1 does not affect pre-meiotic S phase but prevents entry into meiosis I, whereas treatment with 5 µM 1-NM-PP1 blocks pre-meiotic S phase. This indicates that DNA replication in pre-meiotic S phase occurs at a roughly tenfold lower Cdk1 activity than that required for entry into meiosis I, showing that premeiotic stages (G1-S-G2) follows the same quantitative logic as mitosis (Benjamin et al. 2003; Coudreuse and Nurse 2011). Moreover, artificially increasing cyclin-Cdk1 activity in meiosis I, by expressing Clb3 (normally restricted to meiosis II) or Clb2 (not expressed in meiosis), leads to premature sister chromatid segregation, further supporting the existence of distinct Cdk1 activity thresholds that govern meiosis I versus meiosis II events (Carlile and Amon 2008).

### Substrate-level control and phosphoproteomic insights

Together, these findings highlight that meiotic Cdk1 regulation is not only qualitatively distinct from mitosis with specialized and non-redundant cyclins, but also far more constrained in its flexibility. This raises the question of how such a complex regulatory landscape is implemented at the substrate level. Time-resolved global phosphoproteomics has contributed to extensively characterize substrate phosphorylation dynamics in mitosis. Applying this approach to meiosis now allows direct inference of the timing of cell-cycle events, as well as the activity profiles of the kinases and phosphatases involved. Three recently published complementary datasets have mapped the global meiotic proteome and phosphoproteome of *Saccharomyces cerevisiae* with high temporal resolution, offering the first comprehensive view of the meiotic phosphocode.

Wettstein et al., analyzed diploid cells synchronized in G0/G1, induced meiosis and sampled cells at hourly intervals over a 10-h time course covering the full meiotic program. Their data revealed that most proteins and phosphopeptides undergo dynamic changes in abundance or phosphorylation during meiosis, forming sequential waves that reflect stage-specific kinase and phosphatase activities. Notably, numerous proteins of previously unknown function were phosphorylated in a meiosis-specific manner, suggesting potential roles in gametogenesis. This dataset thus provides a broad foundation for identifying critical phosphorylation sites and generating hypotheses about their roles in regulating meiotic processes (Wettstein et al. 2024).

Two additional complementary datasets focused specifically on the meiotic divisions, starting from prophase I, to explore how canonical cell-cycle controls are rewired during meiosis. Using highly synchronous yeast populations, both studies showed that changes in protein abundance are largely restricted to a few key regulators, whereas dynamic phosphorylation events are widespread. In our study, we directly compared meiosis I exit with mitotic exit and recreated a “mitotic exit-like” state in meiosis I. We found that Cdk1 motifs remain largely phosphorylated at the end of meiosis I, whereas most non-Cdk1 motifs are reset by dephosphorylation (Celebic et al. 2024). This observation is consistent with previous genetic evidence showing that mitotic exit regulators are inactive at the MI-MII transition. Indeed, the APC/C^Cdh1^ complex, which promotes cyclins degradation during mitotic exit, is not active between meiosis I and II (Buonomo et al. 2003; Kamieniecki et al. 2005; Ostapenko and Solomon 2024), and the Cdk1 inhibitor Sic1 is not expressed at this stage (Dirick et al. 1998; Benjamin et al. 2003). Residue-specific analysis revealed that threonine-containing Cdk1 motifs TPx(K/R) remained phosphorylated throughout the MI-MII transition, whereas serine-containing motifs SPx(K/R) displayed mixed behavior, with some being dephosphorylated. This strongly contrasts with mitotic exit, where both residues are efficiently dephosphorylated, suggesting meiosis-specific phosphatase regulation (Touati et al. 2018, 2019; Celebic et al. 2024). Markers of phosphatase activity in our phosphoproteomic data indicated partial activation of Cdc14, consistent with the activity of the FEAR but not the MEN pathway at MI-MII (Marston et al. 2003; Kamieniecki et al. 2005; Celebic et al. 2024). This limited activation is sufficient to promote SPB reassembly by half-bridge extension, while excessive or insufficient activity leads to SPB duplication defects (Bizzari and Marston 2011; Fox et al. 2017). Regarding PP2A^Cdc55/B55^, the data showed full activation only in meiosis II (Celebic et al. 2024). Since PP2A^Cdc55/B55^ preferentially targets threonine residues in Cdk1 and Plk1 motifs, its delayed activation likely explains the persistence of threonine phosphorylation at MI-MII (Godfrey et al. 2017; Touati et al. 2019; Kruse et al. 2020; Hoermann et al. 2020). A similar mechanism has been reported in sea urchins, where PP2A^B55^ reactivation, which occurs earlier in this species, preferentially removes TP over SP phosphorylation, suggesting that residue-specific phosphatase control in meiosis is evolutionarily conserved (Swartz et al. 2021). After acute Cdk1 inhibition during budding yeast meiosis I exit to mimic mitotic exit, most phosphosites, including direct Cdk motifs and other kinase consensus sites under Cdk control, adopt a mitotic exit-like phosphorylation state. However, some phosphorylation patterns remain distinct, indicating meiosis-specific regulation (Celebic et al. 2024). Several modifications persisted in a meiosis-specific manner, indicating that local kinase and phosphatase control can override global Cdk1 inactivation. In mitosis, SP sites are phosphorylated and dephosphorylated earlier than TP sites (Godfrey et al. 2017; Touati et al. 2019), yet after Cdk1 inhibition in meiosis I, this hierarchy was lost. Moreover, Cdc5^Plk1^ consensus motifs such as Nx(S/T) displayed distinct behavior compared to mitosis, further supporting the idea that both kinase and phosphatase regulation are developmentally rewired in meiosis (Godfrey et al. 2017; Touati et al. 2019; Celebic et al. 2024). Furthermore, meiosis relies on meiosis-specific kinases such as Ime2, which act alongside Cdk1 to control crucial processes, including the inhibition of DNA replication between the two meiotic divisions. This additional layer of kinase activity, together with other meiosis-specific factors, likely explains why some phosphorylation patterns observed during meiosis do not simply revert to a mitotic exit–like state (Dirick et al. 1998; Guttmann-Raviv et al. 2001; Benjamin et al. 2003; Schindler and Winter 2006; Holt et al. 2007; Phizicky et al. 2018).

In the second study, Marston’s group elegantly demonstrated that some meiosis-specific regulators can rewire the specificity of mitotic kinases, focusing on the mitotic kinase Cdc5^Plk1^ and the meiosis-specific protein Spo13^Meikin^ (Koch et al. 2024). Spo13 (Meikin in mammals) was the first gene identified whose deletion causes cells to undergo only one meiotic division (Klapholz and Esposito 1980). It is primarily known for promoting sister kinetochore monoorientation and protects centromeric cohesin during meiosis I (Katis et al. 2004; Lee et al. 2004; Matos et al. 2008). More recent work has revealed that Spo13^Meikin^ functions as a temporal regulator distinguishing meiosis I from meiosis II, preventing premature expression of specific meiosis II markers and early spore formation (Oz et al. 2022; Rojas et al. 2023). Concerning Cdc5^Plk1^, it recognizes primed phosphosites, S(S/T)P, through its Polo-box domain (PBD). The results obtained by the Marston’s lab suggests that Spo13^Meikin^ binds this same domain, thereby altering substrate recognition in meiosis I (Koch et al. 2024). Their phosphoproteomics revealed that the Spo13^Meikin^-Cdc5^Plk1^ complex preferentially phosphorylates an extended motif, (D/E/N)x(S/T)F, whose phosphorylation is strongly reduced in Spo13^Meikin^ mutants unable to bind Cdc5^Plk1^. This interaction likely shifts Cdc5^Plk1^ specificity, either by modifying its catalytic pocket or by occluding its PBD, thereby favoring the full Plk1 motif (Koch et al. 2024). Because Cdc5^Plk1^ is present in excess, a subset of substrates remains phosphorylated through canonical PBD docking. Moreover, it has been shown that in absence of Spo13^Meikin^, Cdc5^Plk1^ fails to phosphorylate Clb1. The Spo13^Meikin^-Cdc5^Plk1^ complex selectively phosphorylates Clb1 on serine residues, thereby activating it and repressing synthesis of Ama1, an APC/C co-activator specific to meiosis II (APC/C^Ama1^). This mechanism prevents premature meiotic exit (Rojas et al. 2023).

Meiotic phosphoregulation thus illustrates how the “hybrid model” of Cdk1 control can operate under developmental constraints. While quantitative thresholds governing kinase and phosphatase activities are conserved, qualitative layers of regulation through specialized cyclins, docking interfaces, meiosis-specific kinases and regulators, and residue-specific phosphatases ensure the correct order of meiotic events. This specialization reveals the remarkable adaptability of the Cdk network, capable of reusing a universal regulatory logic for distinct cellular outcomes.

Over the past decades, work across yeast and metazoan systems has revealed that Cdk1 control cannot be understood solely in terms of rising and falling kinase activity. Instead, cell-cycle progression emerges from the integration of quantitative changes in Cdk1 activity with qualitative layers of regulation, including cyclin-dependent docking, phosphomotif composition, priming mechanisms, subcellular organization, and the selective action of opposing phosphatases. Together, these features generate ordered phosphorylation and dephosphorylation programs that confer robustness and directionality to the cell cycle. A key emerging result is that phosphatases do not simply reverse kinase activity; they actively shape the temporal readout of Cdk1 signals. Depending on the context, they contribute to quantitative threshold behavior or to qualitative decoding (via residue and motif selectivity, spatial restriction, or docking interactions). The resulting hybrid model reconciles classical views by showing that kinases and phosphatases jointly sculpt the temporal landscape of substrate phosphorylation. Meiosis therefore provides an ideal system to further test and refine this hybrid model. The same core Cdk-phosphatase system must be quantitatively retuned and qualitatively rewired to coordinate two divisions without DNA replication. Looking ahead, a major challenge will be to uncover meiosis-specific regulators that modulate the specificity of kinases and phosphatases. Integrating these studies with global time-resolved phosphoproteomics and predictive modeling of disordered regions could provide a comprehensive view of how the kinase-phosphatase network is dynamically rewired to meet the unique demands of meiotic divisions.

## Data Availability

No datasets were generated or analysed during the current study.
